# The impacts and unintended consequences of the nationwide pricing reform for drugs and medical services in the urban public hospitals in China

**DOI:** 10.1186/s12913-020-05849-4

**Published:** 2020-11-23

**Authors:** Xiaoxi Zhang, Hongyu Lai, Lidan Zhang, Jiangjiang He, Bo Fu, Chunlin Jin

**Affiliations:** 1 Shanghai Health Development Research Center, 1477 Beijing West Rd., Shanghai, 200040 China; 2 School of Data Science, Fudan University, 220 Handan Rd, Shanghai, 200433 China; 3 School of Management, Fudan University, 220 Handan Rd, Shanghai, 200433 China

**Keywords:** Pricing reform for drugs and medical services (PRDMS), Difference-in-difference (DID), Public hospitals, China

## Abstract

**Background:**

Since 2015, China has been rolling out the pricing reform for drugs and medical services (PRDMS) in the urban public hospitals in order to reduce drug expenditures and to relieve financial burdens of patients. This study aims at evaluating the effectiveness of the reform and investigating its positive impacts and unintended consequences to provide evidence basis for further policy making.

**Methods:**

The Difference-in-difference (DID) approach was employed to analyze the reform impacts on the 31 provincial administrative areas in China based on data abstracted from China Statistics Yearbooks and China Health Statistics Yearbooks from 2012 to 2018.

**Results:**

The reform resulted in a decrease of 7.59% in drug cost per outpatient visit, a decrease of 5.73% in drug cost per inpatient admission, a decrease of 3.63% in total cost per outpatient visit and an increase of 9.10% in surgery cost per inpatient admission in the intervention group. However, no significant change in examination cost was found. The reduction in the medical cost per inpatient admission was not yet demonstrated, nor was that in the total outpatient/ inpatient expenses. The nationwide pricing reform for drugs and medical services in urban public hospitals (PRDMS-U) in China is demonstrated to be effective in cutting down the drug expenditures. However, the revealed unintended consequences indicate that there are still significant challenges for the reform to reach its ultimate goal of curbing the medical expenditures.

**Conclusion:**

We conclude that the pricing reform alone may not be enough to change the profit-driven behavior of medical service providers as the root cause lies in the unchanged incentive scheme for providers in the service delivery. This holds lessons for policy making of other low- and middle-income countries (LMICs) with similar health systems set up in the achievement of Universal Health Coverage (UHC).

## Background

Over the past 70 years, China’s health system has undergone vast changes under the profound impacts of the country’s economic reform [1]. In the early years since the People’s Republic of China (PRC) was founded in 1949, the Chinese economy was dominated by central planning and the government took complete charge of its health system [1, 2]. At that time, the government decided on the allocation of health resources and directed the health financing and service delivery. All the health facilities that provided health care, such as hospitals, were solely owned, financed and operated by the government [2]. The government devoted to improving the equity in health service use and impressive improvement in the whole population’s health outcomes were also achieved with only limited health resources [3]. However, the centrally controlled economy led to vast inefficiency and poverty, so China embarked on its economic reform in 1978. Since then, the market force had been performing an increasingly important role in the economy, which also led to the marketization of the health sector in the country [4, 5]. Thereafter, a series of policy interventions were staged to strengthen the market force in the health sector, including the decentralization of public hospital management [6].

As such, the government ceased to fully subsidize the public hospitals so that the public hospitals had to undertake responsibilities for their own profits and losses. At the same time, the government promoted the public hospitals’ autonomy by allowing them to self-manage and determine the pricing of medical services and drugs. The subsidies from the government to public hospital shrank sharply from more than 60% of its total revenue by 1980 to less than 25% by 2008 [7]. That is, instead of relying on the government to finance as before, the public hospitals had to make profits from the drugs and services provided to finance themselves [8, 9]. Consequently, government subsidies, health services and drug sales became the three main sources of hospital’s revenues. In 2012, over 40% of the hospital’s revenues came from drug sales while only approximately 10% came from governmental subsidies [10]. In order to obtain the profit margin, the drugs were allowed to be priced with up to 15% mark-up on the actual purchase price [11]. Moreover, an incentive scheme was introduced to link the physicians’ merit pay, that is a major part of their income, to the hospital’s profits, which would encourage them to prescribe more profitable drug or service [12, 13].

Unlike the successes in the economic reform [14], the marketization of the health sector in China has experienced severe challenges. Once the for-profit management scheme of the public hospital had been established, the motivation of profit-seeking became perverse among health care providers, which led to a significant increase in the revenue of public hospitals and brought about substantial negative impacts [15]. The health care providers are motivated to induce the demand of patients and over-prescribe drugs and diagnostic tests, which resulted in the alarming escalation of health expenditure [16]. From 2007 to 2012, the growth rate of health expenditure (14.9%) far exceeded that of gross domestic product (GDP) (10.2%) [17]. In 2012, the drug expenses accounted for over 50% of the total medical expenditure per outpatient visit and over 40% per inpatient admission [13]. Not only that, extensive over-prescription gave rise to the occurrence of microbial resistance and false-positives diagnostic tests, threatening the quality of health care [18, 19].

Thereupon, complaints from Chinese people on the difficulties of affording quality health care prevailed [20], which were frequently referred to as the lament of “kanbingnan, kanbinggui” or “insurmountable access barriers to health care, insurmountable high health costs” [1]. The outbreak of the SARS epidemic in 2003 further intensified people’s dissatisfaction and thereby the pressing necessities to reexamine the health system [21], which eventually led to the launch of the 2009 reform [22]. With the goal of “everyone has affordable access to basic health care”, the equivalent of $230 billion was committed heavily to the reform between 2009 and 2011 [23]. After several years of efforts, some significant achievements were made, especially in improving health insurance coverage [24, 25]. However, the reform to the public hospitals failed to yield any encouraging progress [1, 26].

In China, public hospitals, which are capable to provide over 80% of the overall inpatient and outpatient services, play the most important role in health care delivery [13]. Therefore, suitable intervention strategies introduced into public hospitals are of crucial importance in the process of an effective health system reform [27], where the aim is to change the profiting scheme of public medical service providers by reemphasizing their mission of improving public welfare instead of earning incomes [28]. Among the various interventions in public hospitals, the pricing reform is regarded as the most substantial instrument, with the core measure as the zero drug mark-up policy, which is to eliminate the up to 15% profit margin that was previously allowed to be added on the actual drug purchase price. In order to ensure the sustainability of the intervention in drug pricing, the pricing of medical services was also adjusted, including raising surgical fee and reducing laboratory fee [29, 30].

The primary aim of the pricing reform for drugs and medical services (PRDMS) is to reduce drug expenditures and thereby to reduce medical expenditures and financial burdens of patients. Meanwhile, by cutting off the economic linkage between drug sales and drug use, the policy also intends to rectify physicians’ behavior in service provision, so as to contribute to the improvement of the quality and accessibility of health care [31]. For the sake of smooth and stable implementation, the government has adopted a step-by-step strategy to push forward the pricing reform. Before intervening into urban public hospitals, the policy had been put into effect in every county-level public hospital (PRDMS-C) by 2015 [32]. Based on the lessons from the implementation in county-level hospitals, some provinces, like Zhejiang and Anhui, took the lead to launch the reform in urban public hospitals. Subsequently, the pricing reform for drugs and medical services in the urban public hospitals (PRDMS-U) has been able to roll out in every public hospital throughout the country as of September, 2017 [33].

A few existing studies have been conducted to evaluate the impacts of PRDMS-C, after it took effect in different areas in China, such as Sanming [34], Zhejiang [35], Hubei [26, 36], Guangxi [37], etc. Most of the studies showed that the reform reduced drug cost whereas its effectiveness in containing medical expenditures was questionable with some unintended consequences [38–43]. For example, through the DID approach, Fu et al. [34] analyzed the public hospital reform in Sanming and showed that the Sanming model was able to reduce drug cost and total medical expenditures without measurably sacrificing the quality or the efficiency of health service provision. It affirmed the effectiveness of the reform in Sanming due to its systematic design and forceful implementation of the policy interventions and justified the nationwide promotion of Sanming model. Using a retrospective pre/post-reform design, Zhang et al. [35] analyzed the questionnaire data from selected county-level public hospitals in Zhejiang from 2011 to 2012 and concluded with a decrease of the supplier-induced demand in drugs but an increase in medical services. Besides, in a study conducted in Hubei, Zhang et al. [26] found that the decrease of drug costs resulted from the reform did not lead to the reduction of personal health spending. As for the nationwide evaluation of the PRDMS-C, Fu et al. [41] conducted a sample investigation to 1880 county-level hospitals across the country and found that the policy resulted in a reduction in drug expenditures together with an increase in diagnostic tests expenditures, which had not measurably contributed to the containing of total health expenditures.

After the reform was completed in county-level hospitals, it is now fully practiced in urban public hospitals in China. Compared with county-level hospitals, the service volumes of urban public hospitals are usually much larger, and the medical services provided are generally more advanced and comprehensive, hence the impacts of reform in urban public hospitals would be even more substantial. Despite some previous studies on the effects of PRDMS-C, the fundamental differences between county-level and urban hospitals limited the generalizability of conclusions of those previous studies on county-level hospitals to urban ones. Although several literatures presented preliminary evaluations in urban cities like Nanjing [42], Beijing [43], etc., the conclusions from these studies can hardly reflect country-level effects of the reform in general as the evidence from the selected locations can hardly be generalized to other areas with different economic and health development background.

In China, a reasonable fee schedule for drugs and medical services has yet been well-established. Detecting the positive influences and unintended consequences of the nationwide pricing reform in urban public hospitals, the most influential player in health service provision in China, is urgent for policy makers to draw lessons from. Therefore, a nationwide impact evaluation of the PRDMS-U with improved methods might be in sore need to provide some empirical evidence to inform further policy-making.

## Method

### Hypothesis

The objectives of the PRDMS-U include four aspects. Firstly, it aims to reduce drug costs through the elimination of the drug mark-up. Secondly, it intends to adjust the cost structure by meanwhile increasing surgical fee and decreasing examination fee. Thirdly, it endeavors to contribute to the reduction of the total medical expenditure. At last, it attempts to rectify the supplier-induced demand and to improve accessibility of medical service for people. The hypothesis of our study is that, the policy has almost achieved the first and the second objectives but not yet realized the third and the fourth ones.

### Data sources and variables selection

We analyze macroeconomic data of 31 provinces/ municipalities collected from China Health Statistics Yearbooks 2012–2018 and China Statistics Yearbooks 2012–2018. The nationwide PRDMS-U was initiated in five provinces in 2015 and then extended to another 14 provinces in 2016. At the end of 2017, all the other 12 provinces were required by the national authority to roll out the reform although some of them were not able to implement the reform until early 2018. Both the timing and the impact of the PRDMS-U vary across provinces, which makes the PRDMS-U be as a “natural experiment”. Given this, we take 2016 as the cut-off point, dividing the observation time into the pilot period (2015–2016) and the non-pilot period (2017–2018). Hence, we define the 19 provinces (Anhui, Fujian, Hebei, Heilongjiang, Hunan, Nei Mongol, Jiangsu, Jiangxi, Liaoning, Shaanxi, Shandong, Shanghai, Tianjin, Zhejiang, Guizhou, Qinghai, Sichuan, Xinjiang, and Yunnan) that initiated the PRDMS-U in the pilot period as the intervention group, while the other provinces (Beijing, Chongqing, Guangdong, Guangxi, Hainan, Henan, Hubei, Jilin, Shanxi, Tibet, Gansu, and Ningxia) are defined as the control group. The idea of grouping based on the timing of policy initiation in DID analysis has been widely used in economics literatures [44–47].

To test the hypothesis, we select several expenditure-related variables to measure the effects of the PRDMS-U, which are total outpatient expenditure, total inpatient expenditure, total expenditure per outpatient visit, total expenditure per inpatient admission, the drug cost per outpatient visit, the examination cost per outpatient visit, the drug cost per inpatient admission, the examination cost per inpatient admission, and the surgical cost per inpatient admission respectively.

Following Fu et al. [34], we also include per capita GDP, per capita public budget revenue, and the ratio of primary industry production to GDP in the analysis as control variables. All the expenditure-related variables are adjusted by 2010 yuan (CN¥) using the CPI and all the variables are estimated in logarithms in this study.

### Model specification

Our empirical strategy is to compare the pre- and post-reform changes between the intervention and the control groups that were both impacted by PRDMS-U. We employ the difference-in-difference (DID) method to evaluate effectiveness of PRDMS-U by using the panel data from 31 provinces/ municipalities during the year period 2012–2018 in China. The basic model (1) is as follows:

1 $$ {Y}_{pt}=\beta \bullet {Intervention}_p\bullet postPRDM{SU}_t+\boldsymbol{\delta} \bullet {\boldsymbol{Control}}_{\boldsymbol{pt}}+{\alpha}_p+{\gamma}_t+{\varepsilon}_{pt} $$

where *Y*_*pt*_ denotes the outcome variables for the p-th province at the t-th year; the dummy variable *Intervention*_*p*_ equals 1 if the p-th province belongs to the intervention group and 0 otherwise; the dummy variable *postPRDMS*_*t*_ equals 1 if the province implemented the PRDMS-U after the t-th year; ***Control***_***pt***_ is a vector of control variables to control unobservable factors; the variable *α*_*p*_ represents the fixed effect used to control those unobserved time-invariant characteristics of the p-th province that may affect the outcome variable; the variable γ_t_ represents the fixed effect used to control the impact of some nation-wide shocks that occur in the t-th year; the term ***ε***_***pt***_ refers to a random error term; the parameter of interest in the difference-in-differences model is the interaction term β between *Intervention*_*p*_ and *postPRDMS*_*t*_; and ***δ*** is a corresponding vector of coefficients for the control variables.

### Comparing the pre-reform trends for the intervention and control group

The difference-in-differences estimator $\hat{\beta}$ is consistent only if differences in outcome medical expenditures between the intervention and the control groups remain constant. Therefore, unparalleled differences derived from the preexisting difference between two groups would bring a potential challenge to the difference-in-differences strategy. To address this problem, we replace the first term in the right side of model (1) by *β*_*t*_ ∙ *Intervention*_*p*_ ∙ *pre*2015_*t*_ ∙ *Year*_*t*_, where the *pre*2015_*t*_ equals 1 if the year is before 2015 and *Year*_*t*_ is a vector of year dummy variables. The coefficient *β*_*t*_ describes the differential change in medical expenditures between the two groups in year *t* before the PRDMS-U. The nationwide PRDMS-U initiated in five provinces in 2015 was then extended to the whole country, hence, annual treatment effects *β*_*t*_ before 2015 can be used to verify the parallel trends. Model (2) is as follows:

2 $$ {Y}_{pt}={\beta}_t\bullet {Intervention}_p\bullet pre{2015}_t\bullet {\boldsymbol{Year}}_t+\boldsymbol{\delta} \bullet {\boldsymbol{Control}}_{\boldsymbol{pt}}+{\alpha}_p+{\gamma}_t+{\varepsilon}_{pt} $$

### Robustness check: controlling for preexisting time trends

Both intervention and control groups may have an increasing trend in medical expenditures after the PRDMS-U because of preexisting time trends or price rigidity, causing the underestimation of the effects of the PRDMS-U in the DID analysis. We extend the model (1) by including an additional term of the time trend *T* to control the potential time trends from pre-reform period and get model (3) as follows:

3 $$ {Y}_{pt}={\beta}_t\cdot {Intervention}_p\cdot {\boldsymbol{YEARpostPRDMSU}}_{\boldsymbol{t}}+\boldsymbol{\delta} \cdot {\boldsymbol{Controal}}_{\boldsymbol{pt}}+\varphi \cdot {Intervention}_p\cdot \mathrm{T}+{\alpha}_p+{\gamma}_t+{\varepsilon}_{pt} $$

where *β*_*t*_ presents the annual reform effects of the PRDMS-U in year t after the PRDMS-U and *γ*_*t*_ indicates year fixed effects controlling for preexisting time trends *φ* ∙ *Intervention*_*p*_ ∙ *T*, where *T* is a vector of time variables.

## Result

### Summary statistics

Table 1 shows the mean values of the observed outcome variables before (period = 0) and after (period = 1) the reform in the intervention group and the control group. The drug cost per inpatient admission experiences a sharp decrease both in the intervention group, 11.80% (Table 1, column 6), and in the control group, 16.20% (Table 1, column 3), after the reform.

**Table 1:** Summary statistics

Variables	Mean of the control group		Mean of the intervention group		Difference		Pr(|T| > |t|)	
	Period 0	Period 1	Period 0	Period 1	Period 0	Period 1	Period 0	Period 1
	(1)	(2)	(3)	(4)	(3)–(1)	(4)–(2)	(7)	(8)
**Medical care cost per outpatient visit**								
Logarithm of total cost (log (CNY))	5.206	5.383	5.274	5.381	0.067	−0.001	0.146	0.975
Increment of the logarithm of total cost (log (CNY))	0.057	0.037	0.048	0.026	−0.009	− 0.011	0.400	0.055*
Logarithm of drug cost (log (CNY))	4.468	4.514	4.535	4.540	0.067	0.027	0.228	0.636
Increment of the logarithm of drug cost (log (CNY))	0.044	−0.007	0.032	− 0.021	− 0.011	− 0.014	0.120	0.120
Logarithm of examination cost (log (CNY))	3.569	3.797	3.663	3.772	0.094	−0.025	0.086*	0.623
Increment of the logarithm of examination cost (log (CNY))	0.066	0.044	0.047	0.037	−0.019	− 0.007	0.415	0.331
**Medical care cost per inpatient admission**								
Logarithm of total cost (log (CNY))	8.917	9.018	8.923	9.024	0.006	0.006	0.912	0.923
Increment of the logarithm of total cost (log (CNY))	0.046	0.025	0.041	0.019	−0.005	−0.006	0.569	0.391
Logarithm of Drug cost (log (CNY))	7.947	7.776	8.014	7.893	0.067	0.117	0.179	0.051*
Increment of the logarithm of drug cost (log (CNY))	0.014	−0.098	0.006	−0.075	− 0.008	0.023	0.375	0.079*
Logarithm of examination cost (log (CNY))	6.441	6.654	6.395	6.590	−0.046	− 0.064	0.305	0.102
Increment of the logarithm of examination cost (log (CNY))	0.073	0.056	0.075	0.058	0.001	0.001	0.898	0.891
Logarithm of surgery cost (log (CNY))	6.038	6.238	6.032	6.258	−0.006	0.020	0.917	0.781
Increment of the logarithm of surgery cost (log (CNY))	0.026	0.096	−0.009	0.101	−0.035	0.005	0.009***	0.838
**Control variables**								
Logarithm of GDP per capita (log (CNY))	10.582	10.765	10.695	10.870	0.113	0.104	0.106	0.208
Logarithm of public budget revenue per capita (log (CNY))	8.416	8.552	8.496	8.629	0.079	0.077	0.398	0.494
Primary industry output as a share of GDP (%)	0.105	0.093	0.100	0.087	−0.005	− 0.006	0.622	0.546

The USD exchange rate for Yuan (USD/CNY) closed at 6.8789 on 2020/1/16

*** *p* < 0.01; * *p* < 0.1

For the intervention group, the annual growth rate of the drug cost per outpatient visit decreases from 3.34% to − 2.05% (Table 1 column 4–5) and that of the drug cost per inpatient admission decreases from 0.66% to − 7.05% (Table 1 column 4–5). The mean of the surgery cost per inpatient service increases from 448.63 Yuan to 565.52 Yuan (Table 1 column 4–5) after the reform in the intervention group, and the growth rate of the surgery cost per inpatient service is 26.06% (Table 1 column 6), significantly higher than 21.96% (Table 1 column 3) in the control group. These results have provided evidence in favor of our hypothesis.

Moreover, in the intervention group, the annual growth rate of the total cost per outpatient visit decreases from 4.98 to 2.69% (Table 1 column 4–5) after the reform, and the annual growth rate of the total cost per inpatient admission decreases from 4.22 to 1.98% (Table 1 column 4–5) after the reform. Likewise, the annual growth rate of the total outpatient cost decreases from 11.86 to 7.52% (Table 1 column 4–5) after the reform and the total inpatient cost decreases from 11.46 to 8.14% (Table 1 column 4–5) after the reform in the intervention group. Additionally, it is noticeable that the examination cost per outpatient visit increases by 10.53% after the reform took into effect in the intervention group.

Based on the t-test results (Table 1 column 7–8), the difference of the control variables between the two groups is not significant, which reveals that the two groups are not heterogeneous in terms of economic and social conditions. Besides, drug cost per inpatient admission has significant differences in period 1 between the two groups (Table 1 column 8), which is consistent with our hypothesis.

**Fig. 1:**
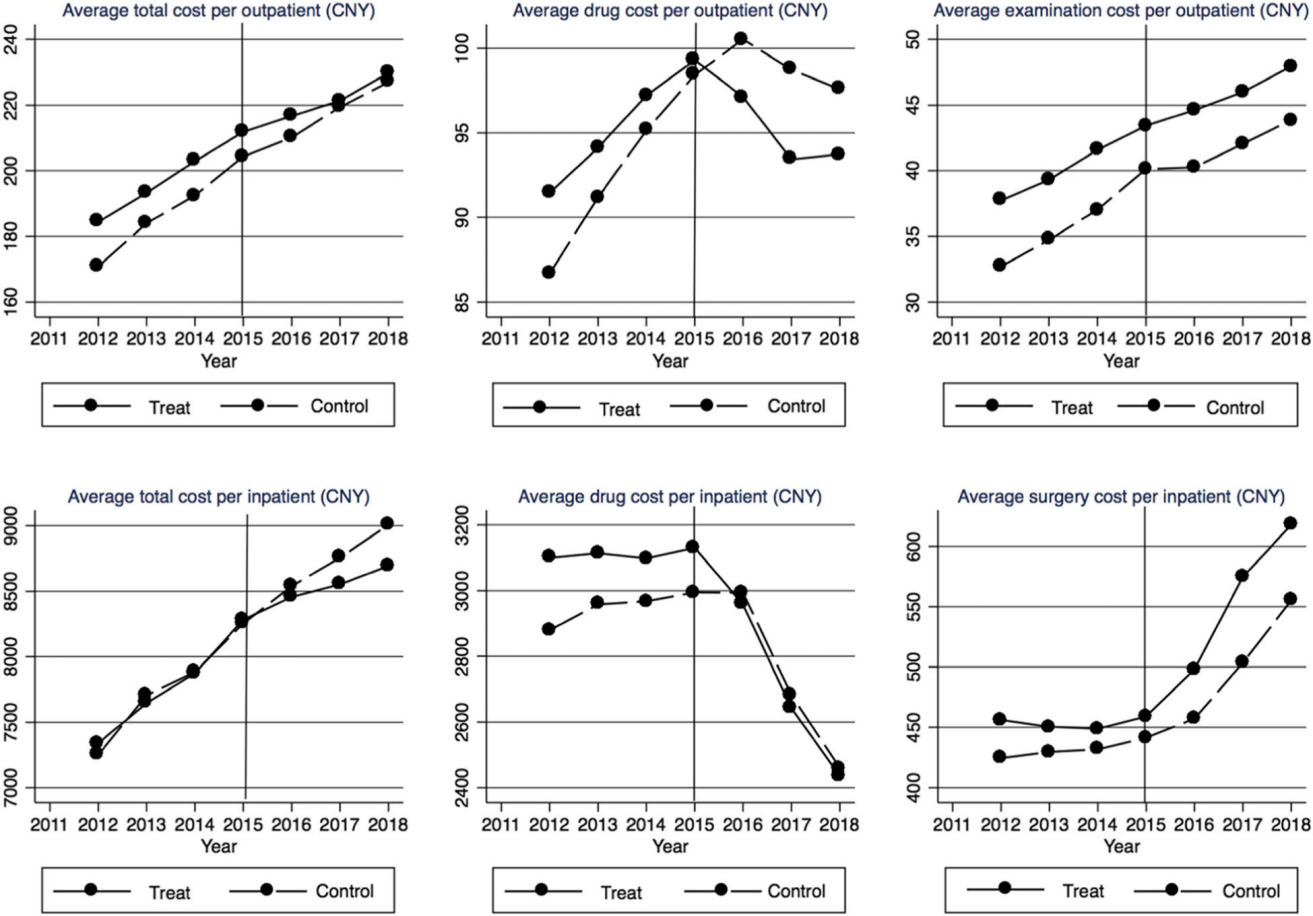
The time trends of outcome variables measuring medical care cost per outpatient visit/ inpatient admission

In order to verify the plausibility of applying DID method (i.e., satisfying the parallel trend assumption), we compare the means of outcome variables between the intervention group and the control group for every year in Fig. 1. All the mean outcome trajectories of the intervention group remain parallel to those of the control group before 2015, although they are separate from each other for all the outcome variables except the total cost per inpatient admission, which demonstrates that there is no heterogeneity trend between the two groups. It is also seen that both the average drug cost per outpatient and the average drug cost per inpatient have a decrease appearing in 2016 and 2017 in the intervention group and the control group respectively. Additionally, the magnitude of increase in surgery cost is shown to be larger in the intervention group.

We also use model (2) to test whether there exists any unparalleled pre-reform time trend between the intervention and control group. The results are shown in Table 2, which reveal no significant differences in pre-reform trends between the intervention and the control groups for most expenditure variables except drug cost per outpatient visit and drug cost per inpatient admission.

### Medical care cost per outpatient visit / inpatient admission

Based on the findings from our parallel trend analysis, we employ the basic DID model (1) for the outcome variables to evaluate the effectiveness of the PRDMS-U. The regression results are shown in Table 3.

**Table 2:** Parallel trend test of outcome variables

	Year	Coef.	Std. Err.	t	P > |t|	95% CI of Coef. (low)	95% CI of Coef. (up)
Total cost per outpatient visit (CNY)	2012	0.0689	0.0327	2.11	0.044	0.0021	0.1357
	2013	0.0250	0.0155	1.61	0.118	−0.0067	0.0566
	2014	0.0328	0.0159	2.06	0.048	0.0003	0.0653
	2015	0.0097	0.0113	0.85	0.4	−0.0135	0.0328
Drug cost per outpatient visit (CNY)	2012	0.1121	0.0326	3.43	0.002	0.0454	0.1787
	2013	0.0789	0.0221	3.57	0.001	0.0337	0.1240
	2014	0.0644	0.0178	3.61	0.001	0.0280	0.1008
	2015	0.0588	0.0150	3.91	0	0.0281	0.0895
Examination cost per outpatient visit (CNY)	2012	0.0920	0.0780	1.18	0.248	−0.0674	0.2514
	2013	0.0421	0.0385	1.09	0.283	−0.0365	0.1207
	2014	0.0218	0.0287	0.76	0.453	−0.0367	0.0803
	2015	−0.0294	0.0374	−0.79	0.437	−0.1058	0.0469
Total cost per inpatient admission (CNY)	2012	0.0380	0.0290	1.31	0.2	−0.0212	0.0972
	2013	0.0058	0.0165	0.35	0.727	−0.0279	0.0395
	2014	0.0155	0.0193	0.8	0.428	−0.0239	0.0550
	2015	0.0187	0.0136	1.37	0.181	−0.0092	0.0465
Drug cost per inpatient admission (CNY)	2012	0.0909	0.0353	2.57	0.015	0.0187	0.1630
	2013	0.0576	0.0276	2.08	0.046	0.0011	0.1140
	2014	0.0516	0.0244	2.12	0.043	0.0018	0.1014
	2015	0.0588	0.0192	3.07	0.005	0.0197	0.0980
Examination cost per inpatient admission (CNY)	2012	0.0123	0.0500	0.25	0.807	−0.0897	0.1144
	2013	−0.0157	0.0304	−0.52	0.609	−0.0779	0.0464
	2014	−0.0105	0.0248	−0.42	0.676	−0.0612	0.0402
	2015	−0.0011	0.0174	−0.07	0.948	−0.0368	0.0345
Surgery cost per inpatient admission (CNY)	2012	−0.0073	0.0610	−0.12	0.906	−0.1319	0.1173
	2013	−0.0450	0.0493	−0.91	0.369	−0.1458	0.0557
	2014	−0.0768	0.0331	−2.32	0.027	−0.1444	−0.0091
	2015	−0.1054	0.0384	−2.75	0.01	−0.1838	−0.0271

Firstly, compared with the control group, the PRDMS-U results in a decrease of 7.50% (=1 − *e*^−0.078^, p < 0.05) in drug cost per outpatient visit (Table 3, column 2) and 5.73% (=1 − e^−0.059^, p < 0.05) in drug cost per inpatient admission in the intervention group (Table 3, column 5), which indicates that the reform policy was effective in cutting the drug expenditure.

Secondly, in the intervention group, the PRDMS-U produces a 3.63% (=1 − e^−0.037^, p < 0.05) decrease of the total cost per outpatient per year after the reform’s implementation (Table 3, column 1), which demonstrates the effect of the policy on decreasing the total medical care cost per outpatient visit. However, the coefficient of the total cost per inpatient admission is not statistically significant (Table 3, column 4), implying that the reform effects on decreasing the total cost per inpatient admission are not yet observable.

Thirdly, the coefficient of the examination cost per outpatient visit or per inpatient admission is not significant (Table 3, column 3 and 6), indicating that the reform has no significant impact on the examination cost. Additionally, the coefficient of the examination cost per inpatient admission is positive (Table 3, column 6), which implies a potential unintended consequence of increasing the examination cost.

Finally, compared with the surgery cost of the control group, that of the intervention group increases by 9.10% (=e^0.087^ − 1, p < 0.05) after the reform (Table 3, column 7), indicating that the reform leads to an increase in surgery cost. Along with the decreased drug cost, it is shown that the reform to some extent promotes the optimization of the fee schedule for drugs and medical services in the urban public hospitals in China.

### Total outpatient/ inpatient expenses

In order to examine the impacts of the PRDMS-U on the total expenditure, we multiply the cost per outpatient visit/ inpatient admission to the number of visits/admissions and obtain the total outpatient/ inpatient expenses as other outcome variables for our analysis. From the results of the DID analysis in Table 4, there is no evidence proving the statistically significant effects of the PRDMS-U on decreasing either the total outpatient expenses or the inpatient expenses. Nonetheless, we find that the coefficients are shown to be negative and the decreasing trend in the annual growth rate of the total expenses can be found in the intervention group from Table 1. It remains to be judged whether the reform has achieved its goals in curbing the upraise of the total medical expenses.

**Table 3:** Impact of the reform on medical care cost per outpatient visit / inpatient admission

Variable	Medicare cost per outpatient visit			Medicare cost per inpatient admission			
	Total cost	Drug cost	Examination cost	Total cost	Drug cost	Examination cost	Surgery cost
DPR	− 0.037**	−0.079***	− 0.027	−0.020	− 0.059**	0.016	0.087**
t-value	−2.22	−3.82	−0.95	−0.91	− 2.19	0.4	2.04
p	0.034	0.001	0.352	0.371	0.036	0.689	0.05
Control variable	√	√	√	√	√	√	√
Year fixed effect	√	√	√	√	√	√	√
Province fixed effect	√	√	√	√	√	√	√
Control variable×T		√					
Control variable×T^2							
Control variable×T^3					√		
No. of sample	217	217	217	217	217	217	217
Adjusted R square	0.8569	0.6378	0.6141	0.7965	0.7691	0.8431	0.6447

*** *p* < 0.01; ** *p* < 0.05

**Table 4:** Impact of the reform on total outpatient/ inpatient medical care expenses

Variable	Total outpatient expenses	Total inpatient expenses	Total expenses
DPR	−0.020	−0.019	−0.019
t-value	−0.71	−0.72	−0.79
p	0.48	0.478	0.438
Control variable	√	√	√
Year fixed effect	√	√	√
Province fixed effect	√	√	√
Control variable×T			
Control variable×T^2			
Control variable×T^3			
No. of sample	217	217	217
Adjusted R square	0.9283	0.9219	0.9315

### Robustness checks

We apply model (3) to control preexisting time trends, and the results are shown in Figures *a* and *b* in Additional file 1, which present robustness checks for drug cost per outpatient visit and drug cost per inpatient admission respectively. The y-axis plots coefficients for the year-specific effects *β*_*t*_ and the year-fixed effects *γ*_*t*_δ_t_. The line for the intervention group indicates the aggregation of *β*_*t*_ and *γ*_*t*_, and the line for the control group indicates *γ*_*t*_. Figure *a* shows that there is a difference of the year-specific coefficients of the drug cost per outpatient visits between the intervention group and the control group occurring after 2014, and the year-specific coefficients of the intervention group are less than 0 from 2012 to 2018. Figure *b* indicates that the year-specific coefficients of drug cost per inpatient admission are more than 0 in both two groups from 2012 to 2018, and a similar difference between the two groups occurs after 2014. The results of model (3) in Figure *a* and *b* in Additional file 1 confirm that the drug expense decreases more significantly in the intervention group than in the control group after the implementation of the PRDMS-U, even when preexisting time trends are controlled.

## Discussion

After the launch of the PRDMS-U, all the urban public hospitals eliminated the drug mark-up and adjusted the prices of medical services; simultaneously, the drug procurement scheme and insurance payment methods were reformed to a certain extent as accompanying policies. Nevertheless, there was a variance in the scope and range of the price adjustment in different areas according to local conditions [33].

In 2015, the General Office of the State Council released *the Guiding Opinions on the Pilot Comprehensive Reform of Urban Public Hospitals* [48]. According to the document, local governments should be responsible for implementing the PRDMS-U; local health administrative departments should be responsible for monitoring the progress of the reform and conducting the progress evaluation for hospitals, the results of which should be substantially linked to the financial subsidies for hospitals and the appointment of hospital directors. Besides, the document also required the percentage of drug expenditure in total medical expenditure to be reduced to about 30%. To meet the requirement, while eliminating the drug mark-up, in fact, public hospitals had to adopt some circumvention measures, such as asking patients to purchase drugs in out-of-hospital pharmacies or raising total expenditures to dilute the share of drug expenditures. In 2019, the General Office of the State Council issued *the Opinions on Strengthening the Performance Evaluation of Tertiary Public Hospitals* [49], in which the requirement for the drug expenditure share had been cancelled.

Based on the conclusions from existing literature, the effects of the pricing reform in public hospitals varied in different scenarios. Fu. et al. [34] evaluated the pilot reforms of public hospitals in Sanming, where the reform achieved tremendous success in reducing drug and medical expenditures, and attributed the effect of the reform there to its substantial alignment of the price adjustment with the reform in the governance structure, payment method and physician compensation scheme. Whereas, some negative impacts of the reform were claimed in more studies conducted elsewhere [35–43]. Tang et al. analyzed antibiotic uses after the reform in Hubei and found that the reform contributed to an increase in the injection of antibiotics, as the hospitals attempted to profit from drug-associated services, such as injections, after the zero drug mark-up policy [36]. Jiang et al. evaluated the reform in Guangxi and suggested that the reform contributed little to the operation efficiency of hospitals and negatively affected clinical quality [37].

Our study contributes to the evidence of the nationwide evaluation of PRDMS-U in China. Through examining the province-level data of 31 areas in China from 2012 to 2018 with the difference-in-difference (DID) approach, our study results indicate that the implementation of the PRDMS-U, with the core measure as the zero drug mark-up policy, is associated with significant reductions in the drug expenses per inpatient admission/ outpatient visit. In other words, the results show that the policy contributes to the reduction in the drug expenditure, which suggests that the policy is on the right track and its preliminary goal has been achieved.

In spite of the striking decrease in drug cost along with the measurable increase in surgical cost per inpatient admission presented, no significant change in examination cost is found, which suggests that the reform objective to adjust the fee schedule for drugs and medical services has not been fully realized despite some positive progress discovered. Moreover, the reduction in the medical cost per inpatient admission is not yet demonstrated, nor is the total outpatient/ inpatient expenses.

These results indicate that, cost-shifting with supplier-induced demand occurs as physicians tend to prescribe more examinations and tests to compensate for the profit loss from drugs, which notably undermines the effectiveness of the PRDMS-U as a whole and results in the failure to reach the ultimate goal of curbing unnecessary expenditures. It is indicated that the pricing intervention alone is unable to relieve the supplier-induced demand. Essentially, despite the elimination of the drug profit margin, the compensation scheme for public hospitals and the payment scheme for physicians remain unreformed. The profits generated from drugs and services still constitute the major part of the revenue of hospitals, a proportion of which makes up the merit pay for physicians. With unaltered economic motivations of suppliers, the reform policy can barely rectify the behavior of health service providers in the concrete sense. This finding is consistent with the conclusion of the national evaluation conducted by Fu. et al. [34] on the reform in county-level hospitals, which also questioned the effects of price control over certain drugs and medical services to curb the health expenditure [41]. It has been pointed out in a number of studies [33, 35–37, 40–43] that the integration of policy interventions was crucial to the effects of the reform and that the piecemeal remedies of the policies could easily lead to circumvention behaviors of health service providers.

The underlying issue is that current China’s health system suffers from serious market failures [50]. Overuse of the market force in service delivery may cause hazards to the equity and affordability of health care [50–54]. Moreover, the particular characteristics of health care market, such as extensive asymmetry of information between suppliers (physicians and hospitals) and demanders (patients), have exerted uncertainty on drawing upon the path of economic reform for health care [16, 55, 56]. Once the for-profit motives get deeply entrenched, the suppliers are prone to induce the demand and push up the price of some profitable drugs or services [57, 58]. Regrettably, the uniqueness of health care market has not been identified thoroughly and the strategy for enterprise management in China’s economic reform has been simply carried over into reforming public hospitals [1].

Interventions from comprehensive scopes should be aligned appropriately to confront the unintended consequences of the PRDMS-U. Above all, from the macroscopic perspective, the role of the government in the health service system should be strengthened to rectify past mistakes [59] in over marketization, including the over-decentralization in the management and development of public hospitals. The government should consider increasing financial subsidies to public hospitals so as to impose greater influence on its economic operation. Moreover, it is of critical importance for the government to be forceful in systematically integrating the policy measures to avoid circumvention behaviors from service providers as a result of the fragmentation and incoordination of governance.

In addition, from the microscopic perspective, the financial incentive mechanisms for suppliers (hospitals and physicians) should be redesigned to positively drive the practice in service provision. On one hand, the financing mechanism of public hospitals should be changed to reduce the dependence of hospital economic operations on drugs and service income. On the other hand, the incentive mechanism for medical staff in public hospitals should be reformed to delink their income from service provision, and meanwhile the public hospital’s authority in using their revenue for staff merit payment should be limited [60]. Besides, a value-based pricing scheme [61, 62] for health care service should be established.

### Strengths and limitations

This study contributes to the knowledge on the nationwide impacts of the pricing reform for drugs and medical services in the urban public hospitals (PRDMS-U). It demonstrates the effectiveness of the reform on cutting the drug expenditure despite some unintended consequences, which reassures the conclusions in some of the previous studies conducted in pilot areas.

As our data were collected from the secondary routine databases, the concerns over the report biases have inevitably limited the quality of the data and caused our incapability to deepen the analysis to the micro level. Actually, we’ve attempted to conduct the propensity score matching (PSM) for our analysis. However, limited by the sample size, the matching process can barely be done sufficiently, which undermines the feasibility of PSM in our scenario. Considering that our study aims at investigating the macro impacts of the policy, it is assumed that some of the individual effects might be offset in the macro-aggregated data, which might be able to reduce the bias caused by the heterogeneity among individuals in the analysis.

Moreover, the reason that few statistically significant difference was obtained might be due to the limited sample size and relatively short follow-up. Hence, continuous monitoring research should also be conducted so as to shed light on the long-term impacts of the reform. Additionally, our analysis focuses on evaluating the impacts of the reform in service expenditures, while further research would be needed to investigate the quality of services.

## Conclusion

Up until now, the PRDMS has been applied to all the public hospitals including county-level and urban ones, which demonstrates the determination of the government in curbing the inflation of medical expenditures and promoting the affordability of health care of people. Our study proves the effectiveness of the policy in decreasing pharmaceutical expenditures. However, the revealed unintended consequences indicate that there are still significant challenges for the reform to confront in the way ahead to reach the ultimate goal.

Several potential solutions are proposed. It is evident that unintegrated policy measures are likely to cause circumvention and the pricing instrument alone should not be enough to change the behavior of providers. Therefore, the combination of interventions in the financing mechanisms for hospitals and physicians is essential. In addition, to enhance the pursuit of social benefits [63], the government should play a fundamental role in service provision and increase financial support to public hospitals. These conclusions hold lessons for other low- and middle-income countries (LMICs) who are also conducting reforms to public hospitals for the optimization of their health service delivery [64, 65].

The policy implementation is never a linear process but full of complexity, which suggests the necessity to conduct continuous monitoring of the policy impacts and perform interventions accordingly.

## Supplementary Information

**Additional file 1: Figure a.** Annual treatment effects for drug cost per outpatient admission controlling for linear time trends. **Figure b.** Annual treatment effects for drug cost per inpatient visit controlling for linear time trends. Parallel trend test of outcome variables.

## Data Availability

The data that support the findings of this study are available from: National Health Commission of the People’s Republic of China. 2012–2018. China Health Statistics Yearbook. Beijing: China Statistics Press. National Bureau of Statistics of China. 2012–2018. China Statistics Yearbook. http://www.stats.gov.cn/tjsj/ndsj/ (accessed 19 Oct, 2020) (in Chinese).

## Abbreviations

PRC: People’s Republic of China
PRDMS: pricing reform for drugs and medical services
PRDMS-U: pricing reform for drugs and medical services in urban public hospitals
PRDMS-C: pricing reform for drugs and medical services in county-level public hospitals
DID: difference-in-difference
LMICs: low- and middle-income countries
UHC: Universal Health Coverage
CI: Confidence Interval
