# Supplementary material for: The impacts and unintended consequences of the nationwide pricing reform for drugs and medical services in the urban public hospitals in China
Source: BMC Health Serv Res. 2020 Nov 23;20:1058. doi: 10.1186/s12913-020-05849-4 (PMC7682084; doi:10.1186/s12913-020-05849-4)
Supplement: Supplementary file 1 — Additional file 1: Figure a. Annual treatment effects for drug cost per outpatient admission controlling for linear time trends. Figure b. Annual treatment effects for drug cost per inpatient visit controlling for linear time trends. Parallel trend test of outcome variables. [file 12913_2020_5849_MOESM1_ESM.docx]

**Appendix**

**Figure a.** Annual treatment effects for drug cost per outpatient admission controlling for linear time trends

The y-axis plots coefficients for the year-specific effect $\beta_{t}$ and the year-fixed effects $\gamma_{t}$ $\delta_{t}$ $\delta_{t}$; the line for the intervention group indicates the aggregation of $\beta_{t}$ and $\gamma_{t}$, and the line for the control group indicates $\gamma_{t}$.

The year-specific coefficients of the drug cost per outpatient visits occurred a difference between the intervention group and the control group after 2014, and the year-specific coefficients of the intervention group were less than 0 from 2012 to 2018.

**Figure b.** Annual treatment effects for drug cost per inpatient visit controlling for linear time trends

The y-axis plots coefficients for the year-specific effect $\beta_{t}$ and the year-fixed effects $\gamma_{t}$ $\delta_{t}$ $\delta_{t}$; the line for the intervention group indicates the aggregation of $\beta_{t}$ and $\gamma_{t}$, and the line for the control group indicates $\gamma_{t}$.

The year-specific coefficients of drug cost per inpatient admission were above 0 in both two groups from 2012 to 2018, and similarly, occurred a difference between the two groups after 2014.

**Table a.** Parallel trend test of outcome variables

|  | Year | Coef. | Std. Err. | t-value | *P* |
| --- | --- | --- | --- | --- | --- |
| Total cost per outpatient visit (CNY) | 2012 | 0.0689 | 0.0327 | 2.11 | 0.044 |
|  | 2013 | 0.0250 | 0.0155 | 1.61 | 0.118 |
|  | 2014 | 0.0328 | 0.0159 | 2.06 | 0.048 |
|  | 2015 | 0.0097 | 0.0113 | 0.85 | 0.4 |
|  |  |  |  |  |  |
| Drug cost per outpatient visit (CNY) | 2012 | 0.1121 | 0.0326 | 3.43 | 0.002*** |
|  | 2013 | 0.0789 | 0.0221 | 3.57 | 0.001*** |
|  | 2014 | 0.0644 | 0.0178 | 3.61 | 0.001*** |
|  | 2015 | 0.0588 | 0.0150 | 3.91 | 0*** |
|  |  |  |  |  |  |
| Examination cost per outpatient visit (CNY) | 2012 | 0.0920 | 0.0780 | 1.18 | 0.248 |
|  | 2013 | 0.0421 | 0.0385 | 1.09 | 0.283 |
|  | 2014 | 0.0218 | 0.0287 | 0.76 | 0.453 |
|  | 2015 | -0.0294 | 0.0374 | -0.79 | 0.437 |
|  |  |  |  |  |  |
| Total cost per inpatient admission (CNY) | 2012 | 0.0380 | 0.0290 | 1.31 | 0.2 |
|  | 2013 | 0.0058 | 0.0165 | 0.35 | 0.727 |
|  | 2014 | 0.0155 | 0.0193 | 0.8 | 0.428 |
|  | 2015 | 0.0187 | 0.0136 | 1.37 | 0.181 |
|  |  |  |  |  |  |
| Drug cost per inpatient admission (CNY) | 2012 | 0.0909 | 0.0353 | 2.57 | 0.015 |
|  | 2013 | 0.0576 | 0.0276 | 2.08 | 0.046** |
|  | 2014 | 0.0516 | 0.0244 | 2.12 | 0.043** |
|  | 2015 | 0.0588 | 0.0192 | 3.07 | 0.005*** |
|  |  |  |  |  |  |
| Examination cost per inpatient admission (CNY) | 2012 | 0.0123 | 0.0500 | 0.25 | 0.807 |
|  | 2013 | -0.0157 | 0.0304 | -0.52 | 0.609 |
|  | 2014 | -0.0105 | 0.0248 | -0.42 | 0.676 |
|  | 2015 | -0.0011 | 0.0174 | -0.07 | 0.948 |
|  |  |  |  |  |  |
| Surgery cost per inpatient admission (CNY) | 2012 | -0.0073 | 0.0610 | -0.12 | 0.906 |
|  | 2013 | -0.0450 | 0.0493 | -0.91 | 0.369 |
|  | 2014 | -0.0768 | 0.0331 | -2.32 | 0.027 |
|  | 2015 | -0.1054 | 0.0384 | -2.75 | 0.01*** |

Note: *** p<0.01; ** p<0.05; * p<0.1
